# Maximization of red pigment production from *Streptomyces* sp. LS1 structure elucidation and application as antimicrobial/antifouling against human pathogens and marine microbes

**DOI:** 10.1186/s43141-022-00452-y

**Published:** 2022-12-21

**Authors:** Nesma A. Hemeda, Ghada E. Hegazy, Soad A. Abdelgalil, Nadia A. Soliman, Dina I. Abdel-Meguid, Samy A. El-Assar

**Affiliations:** 1 Faculty of Science, Alexandria University, Alexandria, Egypt; 2 National Institute of Oceanography & Fisheries (NIOF), Alexandria, Egypt; 3 Bioprocess Development Department, Genetic Engineering & Biotechnology Research Institute (GEBRI), City of Scientific Research & Technological Applications, Alexandria, Egypt

**Keywords:** Pigment, Actinomycetes, Characterization, Antimicrobial

## Abstract

**Background:**

Natural dyes are present in living organisms such as animals and plants and microorganisms such as fungi, bacteria, algae, and yeast. Pigments are fast and easy growth by using cheap components and do not effect by environmental conditions because they required some physical factors like heat, light, and pH and also they have many biotechnological applications such as medical and industrial needs. The natural pigments can act as antimicrobial agents and are used in drug manufacturing. Also, it can be used in the food industry as natural colorants instead of the synthetic colorants due to their safety on human health and low toxicity when emitted into the environment.

**Results:**

A pigmented actinomycetes LS1 strain isolated from El Mahmoudia canal (sediment soil) located in Egypt was microscopically examined and identified as *Streptomyces* sp. by molecular approach. Extraction, purification, and characterization of produced red pigment metabolite like carotenoids related were established based on spectroscopic studies and comparing the data from the literature. Factors (nutritional and physical) influencing red pigmentation by this isolate were investigated through One Variable At Time (OVAT), and then, the optimal levels of the significant key variables were recorded. Also, the productivity yield reached 30 mg of dried purified pigment/gram dry weight. The biological activity of the red product was tested against Gram-positive and Gram-negative marine bacterial pathogens; the recorded antimicrobial activity is more prominent against (*P. aeruginosa* ATCC 9027, *K. pneumoniae* ATCC 13883, *S. aureus* ATCC 6538, *B. subtilis* ATCC 6633 and *E. coli* ATCC 10418) at nearly 0.07 mg mL^−1^ concentration. Also, the tested red pigment showed a positive antifouling activity (AF) against marine microbes; the activity increased by increasing the pigment concentrations from 1 to 3 mg mL^−1^.

**Conclusion:**

The present work focused on the optimization of culture conditions for the production of red pigment by *Streptomyces* sp. LS1; then, the antibacterial activity and antifouling activity of the produced pigments were tested.

## Background

Actinomycetes are a different group of prokaryotic fungus-like filamentous bacteria that are gram-positive, facultative anaerobes and produce early micro colonies composed of thread-like filaments system in their environments with asexual spores singly or in chains [1]. They are widely distributed in various habitats as terrestrial and aquatic habitats [2]. About 90% of actinomycetes members have been found and isolated from the soil and have many industrial, agricultural, and medical applications [3]. Actinomycetes produce many metabolites as pigments which are different in colors such as yellow, green, red, brown, and black [4]. *Streptomyces* is the most active secondary metabolites producer; almost 80% of the natural bioactive substances with medical applications are produced by the *Streptomyces* group [5]. Many studies have been done by researchers to isolate pigment-producing actinomycetes and screen for antimicrobial activity. It has been found that novel antimicrobial substances have been produced by the actinomycetes which are isolated from soil [6]. However, because of the increasing of multidrug-resistant microbes, it is dangerous to the health of the community population. Besides, it has been a significant issue in the treatment of diseases, so we need to discover new compounds that help in solving this problem [7]. So, the present study was targeted to isolate pigment-producing actinomycetes with antimicrobial activity against human and marine pathogens from various geographical regions.

## Methods

### Isolation and screening

Microorganisms used in this study were isolated from different locations in Egypt namely El-Mahmoudia canal, including soil, water, and sediment (in total, 10 samples were collected). Starch nitrate medium (SN) composed of (g L^−1^) Starch 10, K_2_HPO_4_ 2, KNO_3_ 2, casein 0.3, MgSO_4_.7H_2_O 0.05, CaCO_3_ 0.02, FeSO_4_.7H_2_O 0.01, agar 20, pH 7.0 ± 0.2 [8] was applied for actinomycetes isolation, where 25 μg mL^−1^ of Nystatin and 10 μg mL^−1^ Nalidixic acids were added after medium autoclaving to suppress/minimize the growth of fungi and bacteria during the isolation step [9].

### Phenotypic, genotypic, and, physiological characterization of LS1 isolate

The selected isolate (LS1) was characterized morphologically and microscopically via scanning electron microscope (SEM) (Jeol JSM-6360 LA operating at 15 Kv- Central laboratory- the City of Scientific Research and Technological Applications, Alexandria, Egypt) and transmission electron microscope (TEM) (JEM-1400 plus model- electron microscope lab -Faculty of Science Alexandria-University). The method of Kumar et al. [10] was followed to extract the genomic DNA from the selected isolate coded LS1. Thereafter, amplification and sequencing of the *16S rRNA* gene from the genome of the interested isolate was carried out by using a specific forward 16S primer (5′-AGAGTTTGATCMTGGCTCAG-3′) and reverse 16S primer (5′-TACGGYACCTTGT-TACGACTT-3′). The *16S rRNA* sequence was compared with the deposited *16S rRNA* available in the GeneBank database to assess its similarity through Basic Local Alignment Search Tool nucleotides (BLASTn) search (*http://blast.ncbi.nlm.nih.gov/Blast.cgi*); subsequently, the sequence was deposited at GenBank under the accession number: MW585604. Isolate characterization with respect to its ability to produce some enzymes such as lipase, amylase, protease, agarase, and carboxymethyl cellulase was recorded using plate assay as described in the literature [11–15], respectively.

### Pigment production, extraction, and quantification

A culture broth was developed by inoculating a 250-mL Erlenmeyer flask containing 50 mL of Luria-Bertani modified medium (LBM) composed of (g L^−1^) Starch 10, peptone 2.0, YE 4.0, pH; 7.0±0.2 with a freshly prepared three disks (9-mm diameter) of the selected isolate grown in SN agar medium. Then the broth medium was incubated under shaking condition (200 rpm) at 30°C for 7 days. Ethanolic extraction using absolute ethanol 99.9% at 1:1 volume ratio for the produced red pigment (extra and intracellular portions) was carried out individually according to Muthusaravanan et al. [16].

After cultivation, the culture broth was centrifuged at 10,000 rpm for 20 min at 4°C, and then, the supernatant was separated and subjected to extraction directly. While cell pellet was washed thoroughly by saline, then start the extraction after cell disruption through sonication (5×45s) at high-frequency ultrasound (HFU~20 MHz). Successive extractions were carried out until cells were colorless and then centrifuged again. Afterward, the solvent was evaporated at 45°C overnight and the pigment was dissolved in 5 mL of absolute ethanol containing 0.1% of butylated hydroxytoluene (BHT) (phenolic-antioxidant). Samples were kept in dark conditions away from the light source. The extract-colored solution was analyzed by scanning the absorbance in the wavelength region of 200–700_nm_, and then the pigment was quantified by measuring the OD at the wavelength (λ_max410nm_) with the highest absorption and using a standard curve. Extra- and intra-cellular quantification results are combined and expressed as mg%, and cell dry biomass was recorded as well in g%. All measurements were performed in triplex and averaged.

### Identification of the extracted pigment

#### Fourier-transform infrared (FTIR) spectroscopy analysis

The active chemical bonds or functional groups of the dried pigment were identified using Fourier-transform infrared spectrophotometry (Shimadzu FTIR-8400S, Japan) at the Central laboratory for City of Scientific Research and Technological Applications [SRTA-City], Alexandria, Egypt. A mixture of approximately 1 mg of the tested material and 300 mg of pure dry potassium bromide (KBr) was pressed into discs. The measurements obtained infrared spectra between 400 and 4000 cm^−1^

#### Raman spectroscopy

A Raman Senterra instrument (Central laboratory-SRTA-City, Alexandria, Egypt) with a multiwavelength capability operating at 785 nm with a power of 50mW and a wide range of 400–4000 cm^−1^ was used to measure the effect of the excitation wavelength on the pigment spectrum. A laser irradiated an object in an optical microscope (laser spot = 2 μm), and the scattered light from the sample was collected by the optics of the microscope passing through holographic filters, a pinhole, and a monochromator to be detected by a charge-coupled device (CCD).

#### Gas chromatography-mass spectrometry (GC-MS) analysis

This analysis was performed according to a previously reported method by Jerković et al. [17] using an Agilent Technologies GC equipped with a mass selective detector, HP-5MS at the Central laboratory-SRTA-city, Alexandria, Egypt. A 5% phenyl methyl siloxane capillary column with dimensions of 30.0 m × 250 μm × 0.25 μm was used, and helium was used as the carrier gas at 1.0 mL min^−1^. The column T was programmed to initially be 90°C for 1 min, followed by an increase at 8°C min^−1^ to 205°C, then 5°C/min to 240°C, and then 8°C min^−1^ to 300°C. The MS instrument was operated at 70eV. The constituents were identified by a comparison of their mass spectral data with those of standard compounds from the National Institute of Standards and Technology (NIST) Spectral Library.

### Pigment elemental composition

Energy dispersive X-ray (EDX) analysis was carried out at the Central laboratory-SRTA-city, Alexandria, Egypt, to identify the elemental composition of the studied red pigment.

### Medium optimization

#### Medium type and pigment production

For selecting an appropriate basal medium for red pigment production, four different production media namely nutrient broth (NB), LB, LBM, and starch casein (SC) were initially tested, for their ability to support pigmentation by the selected LS1 isolate. The tested media LB, NB, LBM, and SC composed of g L^−1^ [(tryptone 10, YE 5, NaCl 10); (peptone 10, YE 5, NaCl 5); (starch 10, YE 4, peptone 2); and (starch 10, K_2_HPO_4_ 2, KNO_3_ 2, casein 0.3, MgSO_4_.7H_2_O 0.05, CaCO_3_ 0.02, FeSO_4_.7H_2_O 0.01)], respectively. The media pHs were initially adjusted to 7.0 before autoclaving; then, both extra and intracellular pigment yields were monitored and measured in mg% after 7 days incubation time at 30°C and shaking (200 rpm). Also, cell biomass (dried) was measured in g%. The grown cells were separated by filtration, then washed, dried overnight (70°C), then kept in desiccators until constant weight. Finally, the best medium triggering pigment production was chosen as a core production medium in the next experiments in the optimization strategy.

#### OVAT approach and red pigment production

OVAT stepwise approach was applied firstly to determine the effect of T, pH, some salts, different nitrogen, and carbon sources on the production of pigment using LBM as a basal medium at a fixed time (3 or 5 days) and shaking (200 rpm). In all experiments, pigment production-associated growth was detected at λ_410nm_ absorbance for both extra- and intra-cellular portions, then combined and expressed as mg%, and cell dry biomass was recorded as well (g%).

#### Effect of T and pH on pigment production

T effect on pigmentation was studied by incubating the selected production medium with the inoculum of LS strain at different Ts: 25, 30, and 37°C under shaking (200 rpm) for 5 days. Similarly, the effect of pH on pigmentation was carried but at different values of pH (5–9) with increments of 1.0. Cultures were grown under shaking (200 rpm) at 30°C for 5 days using 3 disks inoculum.

#### Effect of salts addition on pigment production

To study the effect of salts on pigment production, the experiment was designed to cultivate the investigated LS1 under the former preferred conditions, but in presence of different salts (NaCl, CaCl_2_, CaCO_3_, and MgSO_4_.7H_2_O) individually added to the unsalted medium at a concentration (%w/v: 0.5 g). Cultures were grown at 30°C, pH 7, and under shaking (200 rpm) for 5 days using 3 disks inoculum.

#### Effect of different nitrogen sources on pigment production

The influence of nitrogen sources on pigment production was determined in the presence of 1% starch and preferred salt. Different formulations for nitrogen sources (organic/inorganic) were tested individually at the following concentrations (%w/v): 0.6 g YE + 0.2 g peptone, 0.6 g YE, 0.6 g peptone, 0.6 g tryptone, 0.6 g malt extract, 0.6 g beef extract, 0.2 g KNO_3_ + 0.4 g YE, 0.2 g NH_4_NO_3_ + 0.4 g YE, 0.2 g NH_4_PO_4_ + 0.4 g YE and 0.2 g NH_4_H_2_PO_4_ + 0.4 g YE, according to the LBM initial composition. The fermentation was carried out under the previously recommended cultural conditions: shaking (200 rpm), T (30°C), pH (7.0), for 3 days using 3 disks inoculum.

#### Effect of different carbon sources on pigment production

The effect of carbon sources on pigment production was estimated by replacing starch (10 g L^−1^) with other various carbon sources at the same concentration in presence of preferred nitrogen source and salt and other cultural conditions. The tested carbon sources include xylose, glucose, fructose, sucrose, mannose, dextrin, galactose, sorbose, ribose, dextrose, gluconic acid, citric acid sodium salt, and glycerol.

### Optimal levels of key variables (nitrogen, carbon, and salt)

Based on the former experiments, YE was found as the best nitrogen source used at 6 g L^−1^. To find the optimal level of this nitrogen source, the previously recognized finest cultural/nutritional conditions were applied in presence of varying levels of YE [g L^−1^] (3, 6, 8, 11, 14, and 17). Also, the final finest recommended conditions were tested in presence of different levels of the best carbon source namely starch then fructose applied at the concentration of 10 g L^−1^. Each sugar was tested individually at levels [g L^−1^] (2, 4, 6, 8, 10, 12, and 14). Additionally, this experiment was repeated but, by using different levels of sodium chloride salt [g L^−1^] (1, 2, 3, 4, and 5) in presence of the best levels of other nutritional components from previous experiments then cultured under the previously recommended cultural conditions.

### Biotechnological applications of red pigment

#### Antimicrobial activity

The antagonistic activity of the extracted red pigments (0.03–0.4 mg mL^−1^) against several marine bacterial pathogens including G_−ve_ (*Escherichia coli* ATCC 10418, *Klebsiella pneumoniae* ATCC 13883, and *Pseudomonas aeruginosa* ATCC 9027) and G_+ve_ (*Bacillus subtilis* ATCC 6633 and *Staphylococcus aureus* ATCC 6538) was tested using the microdilution method [18]. Accordingly, the freshly prepared bacterial suspension of tested pathogens at concentration of 5×10^5^ CFU mL^−1^ (0.5 McFarland) was tested against different concentrations (0.03, 0.07, 0.1, 0.2, 0.3, and 0.4 mg mL^−1^) of a purified red pigment stock solution (1 mg mL^−1^). Two controls, the positive with broad-spectrum antibiotic ciprofloxacin and the negative without any tested compounds, were prepared as well. Then, all prepared tubes were incubated overnight under shaking at 37°C, and the optical density (OD) was recorded. All measurements were performed in triplex and averaged.

#### AF activity

To study the biofouling inhibition, in a 250-mL Erlenmeyer flask, 100 mL of nutrient broth containing glass slide was inoculated with 1.0 mL of seawater for 24 h at 28°C, then pigment was added individually at concentrations 1, 2, and 3 mg mL^−1^. A control flask was prepared using the same conditions without adding pigment. After the incubation period (24 h), the formed biofilms adhered to the surface of the glass slides were stained with 0.4% crystal violet solution for 10 min then washed with water, air-dried, and observed under the microscope [19–21].

### Statistical analysis

All measurements were performed in triplex reactions. The results were expressed as means ± standard deviation which was determined by using Microsoft Office Excel 2013.

## Results

### Actinomycetes isolation and screening the pigment production ability

**Fig. 1:**
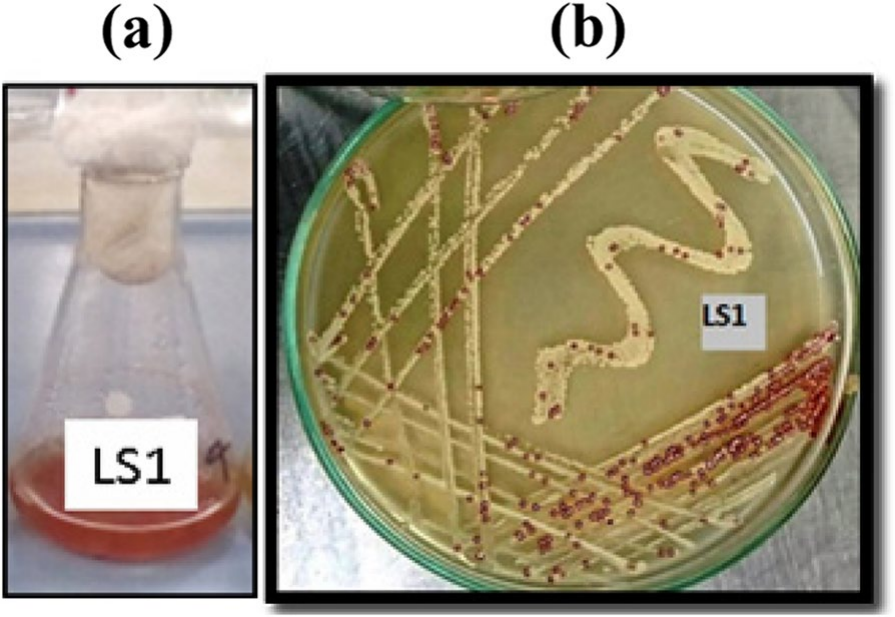
Visual observation for red/orange pigment produced by LS1-isolate in broth medium (**a**) and agar medium (**b**)

Through the isolation program, 50 colonies with different morph types (shapes/colors) were obtained. Among all, only 12 colonies have colors (white, yellow, red, gray, and black) and the others are colorless. Pigment production by visually selected 12 chromogenic isolates was investigated in the LBM medium. The broth medium was inoculated by each isolate and following up color appearance/change was investigated along 7–10 days incubation time at 30°C under shaking (200 rpm) and compared to the control. After screening, the isolate coded LS1 was finally selected to complete this study and recognized as a potential isolate, where it can produce red pigmentation in both solid agar and broth medium in a relatively shorter time (5–7 days) under shaking conditions than other cultures (Fig. 1).

### Characterization and identification of LS1 selected isolate

**Fig. 2:**
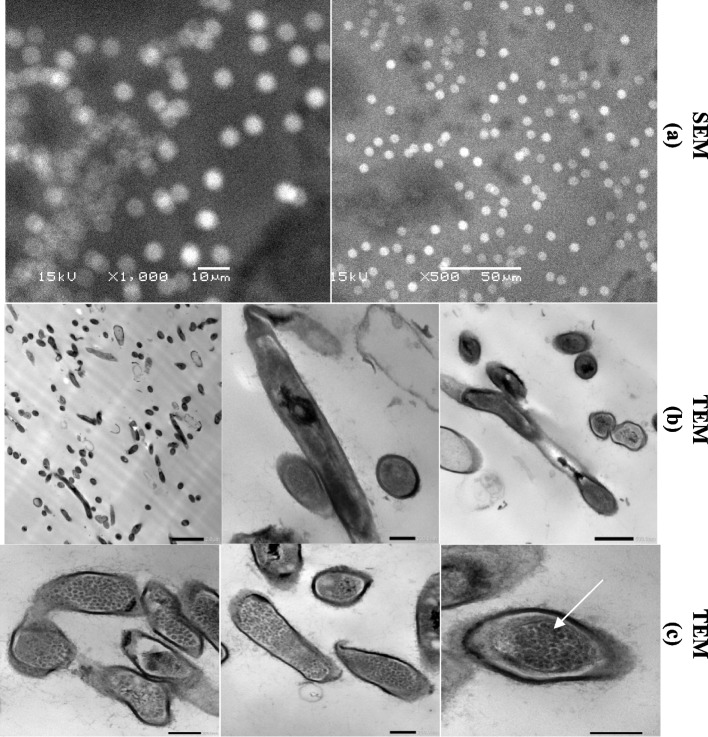
Microscopic examination for LS1 under SEM (**a**) and TEM (**b**), showing spore form (scales bares represent 1μm and 300nm) and the white arrow (**c**) pointed to the pigmented granules inside cells (scales bars represent 500nm and 1000nm)

The selected isolate LS1 appeared +ve in Gram’s stain after examination under the light microscope. A typical radial mycelium characteristic for actinomycetes has appeared under light microscopy for studied LS1 and showed round spore form under SEM (Fig. 2a). Additionally, the stage of spore formation can be easily recognized through the TEM micrograph (Fig. 2b). Cells forming pigment appeared in a curved rod shape and easily recognized the pigmented product inside the cells at different magnification power (Fig. 2c). As seen in the micrograph image, each cell of LS1 isolate appeared to contain pigment granules, which are appeared within the cytoplasm. The isolate (LS1) showed a positive response (able to degrade) towards all tested enzyme substrates: casein, starch, agar, tributyrin, and CMC related to the following enzymes: protease, amylase, agarase, lipase, and cellulase, respectively.

Molecular identification through sequencing, a partial sequence of *16S rRNA* released PCR product was carried out. The obtained sequences (462 bp) were submitted to the BLAST to find homologies with other relevant *16S rRNA* sequences, where they showed 99.57% similarity to *Streptomyces* sp. TD-050 16S ribosomal RNA gene, partial sequence strain (ac: KJ818088.1). Subsequently, the *16S rRNA* of selected isolate LS1 was submitted into GeneBank under *(ac: MW585604)* as *Streptomyces* sp.LS1.

### Red pigment characterization

**Fig. 3:**
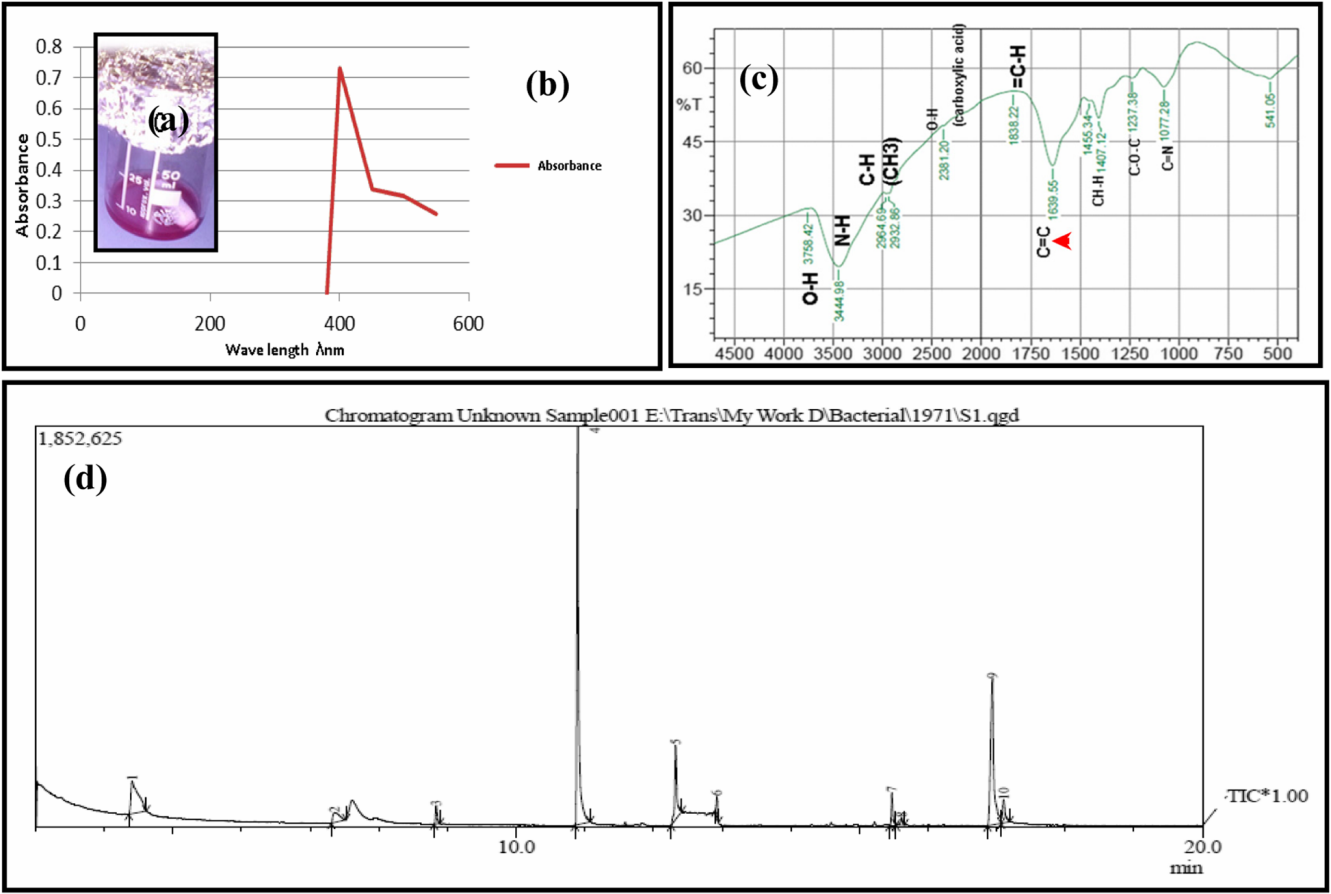
UV-Visible absorption of the partially purified red pigment (**a**) produced by *Streptomyces* sp. LS1 strain showing λ_max_ at 410 nm (**b**), FT-IR spectrum (**c**), and GC-MS chart analysis (**d**)

The partially purified intracellular red pigment extracts (Fig. 3a) yielded by *Streptomyces* sp. LS1 strain was subjected to spectroscopic analyses to elucidate its structure. The chemical structure was elucidated based on Raman spectroscopy, FTIR, and GC-MS analysis.

### Spectral analysis

The obtained dried red pigment was dissolved in distilled water; then scanned (λ_300-700nm_), the highest absorption peak was recorded at 410 nm in the visible light range (Fig. 3b).

### Raman spectroscopy

Results of Raman spectroscopy analysis of the red pigment produced by LS1 have revealed the presence of four regions. It was noticed through Raman spectroscopy, this red pigment has a band at 1300 cm^−1^ which corresponded to CH_3_ umbrella mode, and a band at 1400 cm^−1^ which corresponded to CH_3_ and CH_2_ deformations. Additionally, four bands with relatively equal signal intensities at area 1700–2100 cm^−1^ are corresponded to C=C and C=O bonds.

### Fourier-transform infrared spectroscopy (FT-IR)

The results of FT-IR spectroscopic studies have revealed the presence of various chemical constituents in the pigment extract produced by LS strain (Fig. 3c). The peaks at 541.05 cm^−1^ correspond to alkyl halide stretching frequency. A band at 1077.28 cm^−1^ corresponds to C-OH. The peak at 1237.38 cm^−1^ is assigned to C-O-C. The peaks at 1407.12 and 1455.34 cm^−1^ correspond to CH_3_ and CH_2_ stretching frequency, respectively. The strong peak at 1639.5 cm^−1^ is assigned to the C=C alkene stretching which means that some aliphatic compounds existed in this pigment extract.

### GC-MS analysis

GC-MS analysis for the partially purified LS1 extracted red pigment shown in Fig. 3d indicates the presence of different components. The gas chromatogram shows the relative concentrations of various compounds getting eluted as a function of retention time. The chemical structures of the components are illustrated in Table 1.

**Table 1:** Chemical constituents of ethanolic extract of red pigment produced by *Streptomyces* sp. LS1 strain through GC-MS

ID#	R.Time (min.)	m/z Area	PA	Height	PA%	Compound name	MW g/mol	MF formula	Chemical structure
1	4.409	45.00	885811	97378	42.7	2,3-Butanediol	90.121	C_4_H_10_O_**2**_	
2	7.398	45.00	80479	8919	3.9	2-Propanol, 1,1'-oxybis-	134.17	C_6_H_14_O_3_	
3	8.837	95.00	23607	11973	1.1	Bicyclo[2.2.1]heptan-2-one, 1,7,7-trimethyl-, (.+/-.)-	152.23	C_10_H_16_O	
4	10.895	164.00	522128	236542	25.2	Phenol, 2-methoxy-3-(2-propenyl)-	164.2	C_10_H_12_O_2_	
5	12.326	164.00	133555	69585	6.4	Phenol, 2-methoxy-4-(2-propenyl)-, acetate	206.24	C_12_H_14_O_3_	
6	12.926	149.00	106510	46465	5.1	Diethyl Phthalate	222.24	C_12_H_14_O_4_	
7	15.476	43.00	27299	15017	1.3	n-Hexadecanoic acid	256.43	C_16_H_32_O_2_	
8	15.617	149.00	43492	23068	2.1	Phthalic acid, cyclobutyl isobutyl ester	276.33	C_16_H_20_O_4_	
9	16.940	55.00	207978	63588	10	cis-Vaccenic acid	282.5	C_18_H_34_O_2_	
10	17.103	43.00	43435	10378	2.1	Octadecanoic acid	284.48	C_18_H_36_O_2_	

*RT*, retention time; *m/z*, mass-to-charge ratio; *PA*, peak area; *MW*, molecular weight; *MF*, molecular formula

**Fig. 4:**
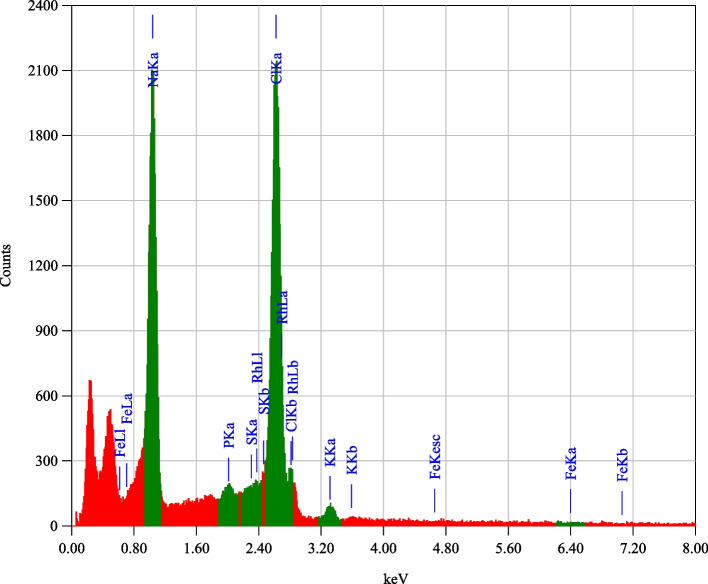
Energy dispersive spectrum (EDS) of *Streptomyces sp*. LS1 red pigment

Ten major compounds have been detected from GC-MS analysis for the red pigment produced by *Streptomyces* sp. LS1 strain, including 2,3-Butanediol; 2-Propanol, 1,1′-oxybis-; Bicyclo[2.2.1]heptan-2-one, 1,7,7-trimethyl-, (.+/-.)-; Phenol, 2-methoxy-3-(2-propenyl)-; Phenol, 2-methoxy-4-(2-propenyl)-, acetate; Diethyl Phthalate; n-Hexadecanoic acid; Phthalic acid, cyclobutyl isobutyl ester; cis-Vaccenic acid; Octadecanoic acid at retention time of 4.409, 7.398, 8.837, 10.895, 12.326, 12.926, 15.476, 15.617, 16.940, and 17.103 min, respectively. The most abundance peak was obtained by 2,3-Butanediol (42.7%) followed by Phenol, 2-methoxy-3-(2-propenyl) – (52.2%) with retention time 4.409 and 10.895 min, respectively, and the least abundance was obtained by Bicyclo[2.2.1]heptan-2-one, 1,7,7-trimethyl-, (.+/-.)- (1.%1) with 8.837 min retention time (Table 1). The chemical structure for ten identified components in red pigment produced by *Streptomyces* sp. LS1 strain is shown in Table 1. The elemental map of the LS1 red pigment was demonstrated by EDX analysis as shown in Fig. 4. The two absorption peaks corresponding to Cl (AT% of 67.12 and mass% of 52.38) and Na (AT% of 30.34 and mass% of 30.70) are most abundant in the sample, while the minor amount of K and P were detected with AT% of 1.62 and 0.92; respectively.

### Start medium for red pigment production

For determination, the most suitable medium is mandatory for red pigment production by *Streptomyces* sp. LS1 strain; four different media namely LBM, LB, SC, and NB broth were tested. The investigated actinobacterium was inoculated as explained in the method section, then incubated under shaking (200 rpm) at (30°C). Based on visual results (color appearance), the maximum red pigment production was achieved in LBM broth after 5 days, while SCA produced less amount of red pigment after 7 days and no red pigment was produced in LB and NB. Quantitative estimation for extracted pigment (extra and intracellular) and biomass yields was determined and is shown in Table 2. According to these results, the highest yield of total pigment was obtained using LBM followed by SCA (~7.2; 7.1 mg%) as well as the biomass yield (~0.24; 0.22 g%), respectively.

**Table 2:** Medium type and red pigment production by *Streptomyces* sp. LS1 strain

Used medium	Extracellular pigment conc. (mg%)	Intracellular pigment conc. (mg%)	Total conc. (mg%)	Biomass (g%)
	**Mean ± SD**			
LBM	1.1618±0.0078	6.0390±0.2137	7.2009±0.353	0.2422±0.0243
SC	1.0866±0.0089	5.9706±0.3010	7.0572±0.1342	0.2202±0.0211
LB	ND	ND	ND	0.1712±0.0165
NB	ND	ND	ND	0.1662±0.0053

*ND*, not detected means no observed color appeared

### Optimization of red pigment production by *Streptomyces *sp. LS1 strain

To screen the bioprocess parameters significantly influencing the red pigment production, the one-variant-at-time (OVAT) method was followed. This allows stepwise optimization, where the best condition was applied in the next one. Thereafter, the optimal level of core variables (best of carbon, nitrogen, and salt sources) was adopted to acquire the best final bioprocess conditions.

### Effect of some physical parameters (T& pH)

It is generally known that incubation T is a crucial factor in any production bioprocess. It was found in this study, a good yield of red pigment was achieved at Ts 30°C and 37°C (~7.5; 7.4 mg%), respectively, while no pigment production at T 25°C was attained. Also, it was noticed that the obtained biomass is higher at T 30 and 37°C rather than 25°C (~0.246; 0.244; 0.1722 g%), respectively. Also, the current work explored that varying the pH of the selected LBM medium tends to play a key role in pigment production and microbial growth. The tested *Streptomyces* sp. LS1 strain was cultivated at different initial pH values (5.0–9·0), then incubated under shaking (200 rpm) and at optimal T (30°C). The results indicated that the highest level of pigmentation and biomass were obtained at neutral (~7.7; 0.26 mg%; g%), respectively, while a little reduction in pigment and biomass production (~7.0; 0.24 mg%; g%), respectively, were attained at pH 6.0. Also, it is noticed that pigment production declined at acidic pH value (pH- 5) (5.7 mg %), while the alkaline pHs (8 and 9) did not support the pigment production at all.

### Effect of nutritional parameters (salt addition, nitrogen, and carbon sources)

To assign a suitable salt source for the red pigment production, the investigated *Streptomyces* sp. LS1 strain was cultivated under the previously recommended medium and cultural conditions but in presence of the following salts (NaCl, CaCl_2_, CaCO_3_, and MgSO_4_) which were individually added to the medium, then compared to the unsalted medium. Among the tested salts, both NaCl and MgSO_4_ at the concentration (3 g L^−1^) enhanced the pigmentation by 1.5- and 1.1-fold, respectively compared to the unsalted medium. The highest level of pigment formation and biomass were detected (~11.7 mg%, 0.26 g%) in presence of NaCl followed by MgSO_4_ (~8.4 mg%, 0.24 g%); however, there is no pigment production was recognized in presence of either CaCl_2_ or CaCO_3_. Further, the organic nitrogen source namely YE showed a maximum production for both total red pigmentation and biomass (~9.5 mg%, 0.26 g%) among all the tested nitrogen sources, followed by YE and peptone (in a mixture) (~13.4 mg%, 0.27 g%). On the other hand, a lower red pigment production was obtained by using either peptone or tryptone in individuals (~8.3 and ~6.1 mg%), respectively, while using beef extract or malt extract did not support either pigmentation or growth as shown in Table 3. By the addition of an inorganic nitrogen source to YE (ratio 1:2), the least pigmentation yield (~7.1 mg%) was obtained through the combination of YE+NH_4_H_2_PO_4_; however, the highest (8.1 mg%) was obtained through the combination of YE+NH_4_NO_3_ as shown in Table 3. Accordingly, still, the selection of YE only (as a nitrogen source) is the best for pigment production conditions. Therefore, it was applied to complete the optimization strategy by testing different carbon sources. Out of the examined 15 carbon sources, the highest level of pigment formation by the studied LS1 strain was detected in the presence of fructose, dextrin, and starch (~17, ~14.2, and ~13.6 mg%) followed by lactose, mannose, and galactose (~13 mg%), and a moderate level (~10-5 mg%) of pigment formation was detected by sorbose, ribose, dextrose, gluconic acid, and sucrose and no pigment production in presence of glucose, glycerol, xylose, and citric acid as carbon source (Table 4).

**Table 3:** Nitrogen sources and red pigment production by *Streptomyces* sp. LS1 strain

	Extracellular pigment conc. (mg%)	Intracellular pigment conc. (mg%)	Total (mg%)	Biomass (g%)
	Mean ± SD			
**Using organic nitrogen source**				
Y.E+Peptone	1.5552±0.09827	7.9543±0.1474	9.5095±0.0891	0.2598±0.0067
Y.E	2.2187±0.1833	11.1693±0.1398	13.3880±0.1322	0.2722±0.01431
Peptone	1.2986±0.0846	7.0138±0.0943	8.3124±0.0472	0.237±0.0098
Tryptone	1.0661±0.0759	5.0814±0.0843	6.1474±0.0583	0.236±0.0089
Beef-extract	ND	ND	ND	0.1664±0.0078
Malt-extract	ND	ND	ND	0.1726±0.0093
**Using mixed nitrogen source (organic/inorganic at 1:2 ratio)**				
Y.E+KNO_3_	1.250765	6.6718±0.0743	7.9225±0.0398	0.2468±0.0091
Y.E+NH_4_NO_3_	1.250765	7.0138±0.0941	8.2645±0.0593	0.2462±0.0097
Y.E+NH_4_PO_4_	1.134479	6.5521±0.0067	7.6865±0.0469	0.2382±0.0087
Y.E+NH_4_H_2_PO_4_	1.021612	6.1074±0.0049	7.1290±0.0548	0.2378±0.0087

*ND*, not detected means no observed color appeared

**Table 4:** Carbon sources and red pigment production by *Streptomyces* sp. LS1 strain

Tested carbon source	Extracellular pigment conc. (mg%)	Intracellular pigment conc. (mg%)	Total (mg%)	Biomass (g%)
	**Mean ± SD**			
Starch	2.0921±0.0336	11.5113±0.0567	13.6034±0.0811	0.509±0.0043
Dextrin	2.2871±0.0841	11.9218±0.0711	14.2089±0.0677	0.4842±0.0073
Fructose	2.7762±0.0727	14.2304±0.0432	17.0066±0.0832	0.5358±0.0061
Galactose	2.0921±0.0339	11.4258±0.0598	13.5179±0.0891	0.484±0.0049
Mannose	2.0921±0.0338	11 .4259±0.0599	13.5179±0.0899	0.4732±0.0043
Lactose	2.0921±0.0317	11.4943±0.0567	13.5863±0.0811	0.4872±0.0067
Sorbose	1.4320±0.0369	9.1001±0.0139	10.5321±0.0567	0.2576±0.0042
Ribose	1.4251±0.0895	6.8940±0.0876	8.3193±0.08265	0.255±0.0040
Dextrose	1.3671±0.0564	6.8940±0.0875	8.2611±0.0399	0.2526±0.0063
Gluconic acid	1.0763±0.0357	6.2613±0.0518	7.3377±0.0867	0.2326±0.0033
Sucrose	0.8711±0.0249	4.5170±0.0516	5.3881±0.0757	0.2014±0.0023
Glucose	ND	ND	ND	0.1682±0.0028
Glycerol	ND	ND	ND	0.1562±0.0038
Xylose	ND	ND	ND	0.1322±0.0040
Citric acid	ND	ND	ND	0.1318±0.0044

*ND*, not detected means no observed color appeared

### Key variables (YE, fructose, and NaCl) optimal level

By testing different levels of YE 0.3–1.7 g (w/v%), the results explained that YE at concentration 0.3 g% was optimal for pigmentation and biomass production (~16.3 mg%; ~0.5326 g%). Also, to find the optimal level of the selected carbon source (fructose), the experiment was repeated under the former preferred recommended cultural and nutritional condition but in presence of different concentrations of fructose 0.2–1.4 g (w/v %), then the total pigmentation and biomass were recorded. The results explained that significantly the highest level of pigment formation and biomass (~17.4 mg%; ~0.62 g%) was obtained at %w/v (0.8 g) of fructose. Finally, to achieve the highest yield of pigmentation, different concentrations (0.1–0.5g w/v %) of NaCl (salt) were tested; it was found that the highest level of pigment formation and biomass (~20 mg%; ~0.65 g%) was attained at 0.3 g (w/v %) of sodium chloride.

### Final formula of the optimized medium

Based on OVAT optimization results and key variables optimization experiments, the formula of the optimized medium (g L^−1^) is as follows: YE, 3; fructose, 8; NaCl, 3; pH, 7; cultivation under shaking, 200 rpm; T, 30°C; and incubation time, 72h.

### Antibacterial/anti-biofouling activities

**Fig. 5:**
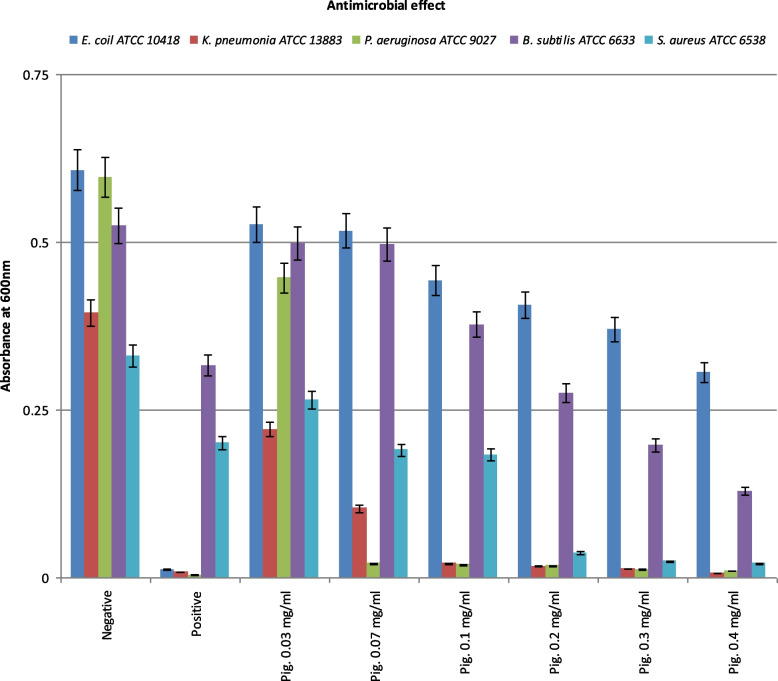
Antimicrobial activity of LS1 *Streptomyces* sp. pigments extract against five species of pathogenic microbes.

**Fig. 6:**
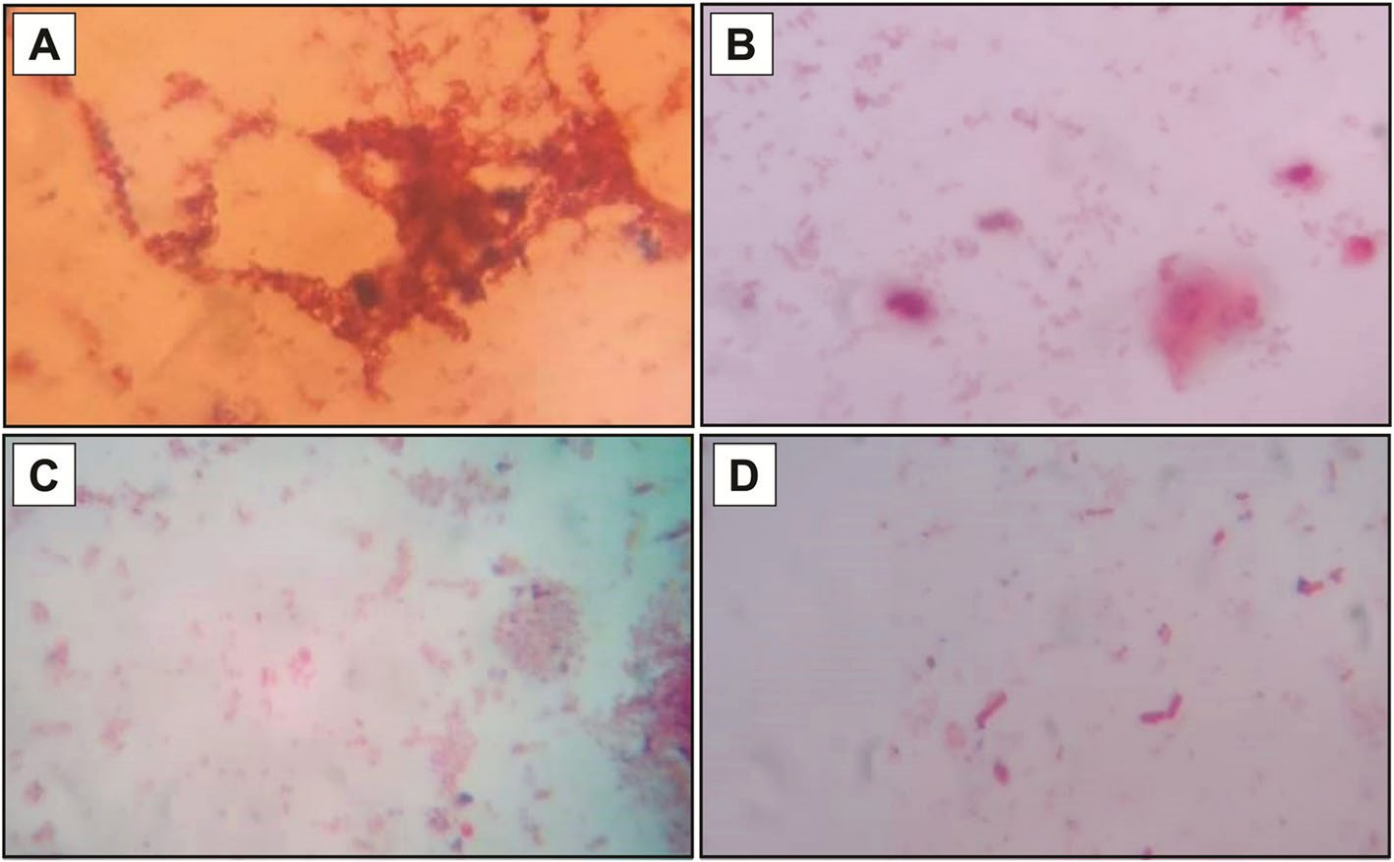
Effect of LS1 *Streptomyces* sp. strain pigments extract on biofilm formation **A** in the absence of the extract, **B** in the presence of 1mg/ml of the pigments, **C** in the presence of 2 mg mL^−1^ of the pigments, **D** in the presence of 3mg mL^−1^ of the pigments

The antimicrobial activity of LS1 carotenoid pigment was studied against five species of marine bacterial pathogens as shown in Fig. 5. It was found that *P. aeruginosa* ATCC9027, *K. pneumoniae* ATCC 13883, and *S. aureus* ATCC 6538 were more susceptible to carotenoid pigment extracted from LS1 strain than *E. coli* ATCC 10418 and *B. subtilis* ATCC 6633. In addition, microscopic examination for the dried glass cover indicates that the tested LS1-pigmented products inhibited biofouling formation. A lower count of bacterial biofilms on the treated panels (treated glass slides) was observed against the untreated ones. By increasing pigment concentration, 1–3 mg mL^−1^, the number of adhered bacteria decreased, i.e., the pigment has a remarkable valuable effect on biofouling reduction (Fig. 6).

## Discussion

Recently, the world has tended to use colorants from natural sources owing to the many problems resulting from the use of industrial colorants in many industries, such as food and medicine [22]. Microbial pigments are of interest because they are often more stable and soluble than those from plant or animal sources [23]. Microorganisms can grow rapidly, which could end in high productivity, and will produce a product throughout the year. Thus, the food industry has become increasingly inquisitive about the utilization of microbial technology to supply colors to be utilized in foods. It is going to help to beat the growing public concern over the adverse health effects of the addition of synthetic colors in food products. Aside from the health benefits, natural colorants will also be a boon to biodiversity since toxic chemicals emitted into the environment when creating synthetic colorants may be eliminated. These natural colorants are utilized in baby foods, cheese, fruit drinks, vitamin-enriched milk products, and some energy drinks. In this way, natural colors may serve the twin purposes of appealing to the eye and providing health advantages for probiotic bacteria in food, in addition to being environmentally beneficial [24].

There are some kinds of micro-organisms that have the pliability to supply pigments in high yields, including species *Streptomyces* [25]. *Streptomyces* ssp. can produce an array of pigments having antibacterial or antifungal properties which are applied for human pharmaceutical use [26].

This study focused on the isolation of actinomycetes from samples collected from the El-Mahmoudia canal, in Egypt in diverse forms (soil, water, and sediment). A typical isolation protocol for actinomycetes was followed as described by Rahman [27]. Finally, in total, 50 colonies with different morphology types (shapes/color) forming pin-point shapes were obtained; among them, 12 are colored. Among these 12 chromogenic cultures, the isolate coded LS1 was selected, where it produced a visible amount of red/orange pigmentation in both agar and broth media. A well-developed radial mycelium is characteristic of classic actinomycetes; this mycelium may be split into substrate mycelium and aerial mycelium in accordance with shape and performance [28]. One of the most diverse groups of Gram-positive bacteria, Actinobacteria has the highest G+C content and shows the most complex morphological differentiation, based on filamentous levels of the organization, much like a filamentous fungus. A variety of actinobacteria may generate complex structures such as spores, spore chains, sporangia, and sporangiospores, among other characteristics [29]. Till now, most actinobacteria are characterized and classified on their morphology in the first place. The morphological characteristics are still one of all the foremost basic indexes which give in-depth information on a taxon. Thus, morphological properties of studied isolate LS1 were intensively characterized through several microscopies, picked micrographs, colony characteristics, areal hyphae, and spore formation. LS1 strain showed a typical radial mycelium characteristic for actinomycetes under light microscopies and showed round spore formation under light microscopy and SEM. The stage of spore formation was recognized through the TEM micrograph. Additionally, cells forming pigment appeared in a curved rod shape where it was easily recognized the granulated pigmented products inside the cells at different magnification power.

Over the years, GenBank information based on the *16S rRNA* gene has been constructed and it had been successfully utilized for the differentiation of bacteria [30]. Genotypic identification emerged as a complement to determine phenotypic methods. Genotypic identification of bacteria often entails using conserved sequences across phylogenetically informative genetic targets, such as the small subunit *16S rRNA* gene [31]. Also, the *16S rDNA* sequence comparison has been used as a strong tool for establishing phylogenetic and evolutionary relationships among organisms [32]. So, the *16S rDNA* of the studied LS1 isolate was amplified, sequenced, and compared to the general public data in GeneBank. It showed 99.57% sequence homology to *Streptomyces* sp. TD-050, accordingly the studied bacterium, was designated as *Streptomyces* sp. LS1. Subsequently, *16s rRNA* of LS1 was deposited in GenBank with accession number MW5856041.

Pigments are usually categorized as secondary metabolites, produced mostly inside the cells. So, sonication was applied for LS1 cell disruption, followed by repeated ethanolic extraction (v:v ratio 1:1). There are several laboratory-based approaches for cell disruption, including ultrasonic cavitation, which uses 15–20 kHz ultrasound waves to create an acoustic pressure wave that breaks apart the cell membrane [33]. Similarly, many researchers use sonication for cell rupture and liberating bioactive products like the laccase enzyme [34]. Also, the osmotic shock was used for liberating the pigmented products, especially just in the case of halophilic archaea bacterial strains as reported by Hagazy et al. [21]. Additionally, many protocols for pigment extraction are usually followed using different solvents like hexane, benzene, chloroform, methanol, acetone, ethanol, or mixed solvent [35]. However, during this study, successful extraction for LS1 pigment was observed by using the organic solvent ethanol with a purity of 99.9% (at a volume ratio 1:1). Similarly, Muthusaravanan et al. [16] used the organic solvent (ethanol) for pigment extraction at a volume ratio of 1:1 in a successful way. The carotenoid pigments are most ordinarily found in nature; their absorption is mostly localized within the range 400–500 nm [36]. Additionally, the wavelengths 400, 470, and 500 nm were identified as having the greatest pigment absorption, reflecting the absorption maxima for yellow, orange, and red pigments, respectively [37]. The pigment isolated during this work showed a light-weight absorption characteristic at 400–410 nm, it indicating that might be associated with the carotenoids group of pigment [36].

It was stated that both Raman spectroscopy and infrared spectroscopy are effective in analyzing the total fingerprint of carotenoids. IR spectroscopy provides information about the outer structures, whereas Raman proved with a signature of the inner structure with minimal information about the outer structure. The combined use of Raman and infrared spectroscopy could be a good way to see a full spectral fingerprint of carotenoids. Accordingly, further characterization of the studied LS1 pigment was applied using these advanced and fast analytical tools (Raman and FT-IR-spectroscopy), and so GC-MS was used for elucidation of the chemical structure. It was noticed through Raman spectroscopy, the studied red LS1 pigment features a band at 1300 cm^−1^ which corresponded to CH_3_ umbrella mode, and a band at 1400 cm^−1^ which corresponded to CH_3_ and CH_2_ deformations. This finding was virtually identical to that reported by Kushwaha et al. [36], who hypothesized that strong Raman bands in the region of 1511–1530 cm^−1^, 1153–1159 cm^−1^, and 1003–1010 cm^−1^ indicate bacterial carotenoids. Furthermore, Hegazy et al. [21] discovered the presence of three regions with two strong and weak signal intensities for orange carotenoids in the pigment produced by *Natrialba* sp., with a robust band at 1380 cm^−1^, which corresponds to the CH_3_ umbrella mode, and a weak band at 1883 cm^−1^, which appreciates C=C bonds. Additionally, four Raman bands of LS1 pigment with relatively equal signal intensities at area 1700–2100 cm^−1^ are corresponded to C=C and C=O bonds; these confirm the relatedness of the studied LS1 pigment to the carotenoids group.

FTIR, on the opposite side, could be a technique that is employed to get a spectrum, emission, and photoconductivity of a solid, liquid, or gas. FTIR that operates within the mid-infrared region (4000–400 cm^−1^) may be a powerful tool for qualitative analysis of fats, oils, and palm carotene [38]. Hence, the studied pigment was subjected to FT-IR spectroscopy analysis, it was found several peaks at 541.05 cm^−1^ correspond to haloalkane stretching frequency, at 1077.28 cm^−1^ corresponds to C-OH, and at 1237.38 cm^−1^ is assigned to C-O-C. Also, peaks at 1407.12 and 1455.34 cm^−1^ correspond to CH_3_ and CH_2_ stretching frequency, respectively. The strong peak at 1639 cm^−1^ is assigned to the C=C alkene stretching which suggests that some aliphatic compounds existed in LS1 pigment extract; this was verified through GC-MS as well. The presence of such spectral bands confirmed that the studied LS1 pigment resembles a high extent to the carotenoids, especially peaks at 1407 cm^−1^ which appear that related to the bending vibration of methylene –CH_2_ as recorded by Hosseini and Jafari [39] when using beta-carotene standard. The height that appears at 2932 cm^−1^ of the studied pigment may well be attributed to the β-ionone ring of beta-carotene because of the C-H, (–CH_3_) symmetrical bending [36].

Optimization of media components, culture parameters, and strain improvement are essential tools to improve the performance of the bacterial system which helps to extend the yield of its products economically. This system has been applied for the optimization of various process parameters and medium composition [40]. Submerged pigment production is riddled with several biotechnological processes and environmental parameters such as temperature, pH, salt, nitrogen, and carbon sources [41]. It is vital to control them in industrial bioprocesses. Metabolically, the implications of them are associated with changes within the activities of proteins; therefore, the culture conditions can control some activities like cellular growth, production of primary and secondary metabolites, fermentation, and so the oxidation processes of the cell. Within a few investigations, the ideal conditions for *Streptomyces* ssp.’s red pigment production have been explored [42].

Microorganisms’ growth and development are greatly influenced by environmental parameters such as temperature, which also has an impact on a variety of biosynthetic processes such as pigment production. Also, the biosynthesis of a pigment is significantly tormented by the physiological parameter and temperature [43]. To seek out the optimal temperature for pigment production by the investigated LS1 strain of *Streptomyces* sp., it had been cultivated under various temperatures (25–30–37°C). As a result, it was discovered that the ideal temperature for pigment production is 30°C. *Streptomyces* sp. strain LS1 is thought to have a favorable physiological characteristic that allows it to maintain this ideal temperature. This observation was in agreement with studies with *Monascus* cultures, red pigment production was highest at 30°C, and decreased at temperatures beyond 40°C in the midst of a rise within the production of yellow pigments [44]. In other studies the growth conditions for red pigment production by a novel strain of *Bacillus* sp. located at 34°C, which is the optimum for pigment production by this novel isolate, while *Pseudomonas aeruginosa* strain was reported to supply maximum pigment production optimally at 37° [45].

Pigment production by an organism is affected largely by the pH of the medium within which the microorganism is grown. Slight changes in pH can even alter the shade of color produced [46]. The influence of pH on the assembly of red pigment by LS1 was studied at different pH values starting from 5 to 9 pH. The results showed that maximum production of red pigment by investigated LS1 strain occurred at pH 7. However, the acidic (pH 5) showed along with the all-time low synthesis of the pigment, and no pigment at all was found at basic/alkaline pH (8 and 9). This agreed with studies that found a maximum melanin activity at neutral pH 7, while further increase in pH reduced the melanin by the actinomycetes isolate in starch nitrate medium [47]. Inversely, Mortazavian et al. [48] found that the most effective results for the assembly of yellow pigments were obtained at initial pH values from 3.0 to 3.5, while the finest results for the assembly of red pigments were reached at pH levels between 7.0 and 7.5.

The effect of salt on pigment production was evaluated through the individual addition of the subsequent salts to the medium: NaCl, CaCl_2_, CaCO_3_, and MgSO_4_.7H_2_O at concentrations 3 g L^−1^ against unsalted medium (control). The significantly highest level of pigment formation by studied LS1 strain was detected in presence of NaCl, followed by MgSO_4_, while no pigment production in the presence of CaCl_2_ or CaCO_3_. This observation was in agreement with studies of melanin production from Actinobacterium *Nocardiopsis alba* MSA10, the best melanin production (3.4 mg mL^−1^) has been obtained at 2.5% of salinity [49]. Another study found CaCl_2_ (10 mM) slightly enhanced the pigment production by *Paecilomyces sinclairii* [50]. According to Chaskes and Tyndall [51], there is a correlation between nitrogen sources and pigment production. This study tested the effect of various nitrogen sources on pigment production by LS1 strain in the presence of 1% starch (carbon source). Different formulations for nitrogen sources (organic/inorganic) were tested individually or in combinations. The organic YE alone has shown the maximal production of both pigment and biomass (growth) (13.38 mg% and 0.272 g%), among all tested formulations, followed by a mixture of YE and peptone, while low production values upon lonely using peptone or tryptone. Limited growth and pigmentation were found when using mixed organic and inorganic nitrogen source. However, there were no growth and no pigment just in case of using either beef extract or malt extract alone. This observation was in agreement with studies done by Budihal et al. [52]; they screened nine nutrient parameters and found that YE is the finest for maximum carotenoid production. Another study has shown that organic nitrogen sources promote greater mycelial growth as compared to inorganic nitrogen sources. Pigment production was stimulated by meat peptone, casein peptone, the peptone–YE combination, and corn steep powder, but red pigment synthesis was severely hindered by soy peptone and malt extract [53].

A stimulatory effect of carbon source on LS1 pigment production was estimated through replacing starch (10 g L^−1^) with other various carbon sources at the identical concentration in presence of a preferred nitrogen source from the previous experiment. Among the tested carbon sources, the highest level of pigment formation by studied strain LS1 was detected with fructose, dextrin, and starch followed by lactose, galactose, and mannose, and a low level of pigment formation was detected by sorbose, ribose, gluconic acid, and sucrose, and no pigment production in glucose, glycerol, xylose, and citric acid. This observation was in agreement with El-Batal and Al Tamie [54]; they found starch is the best source for the highest production of melanin by *Aspergillus oryzae*. Similarly, the same results were found by Venkatachalam et al. [55], who reported that starch was the foremost effective carbon source for the assembly of melanin, followed by glycerol and fructose. However, Hewedy and Ashour [49] reported that *Kluyveromyces marxianus* and *Streptomyces chibaensis* produced brown pigment in presence of xylose as a carbon source.

Since YE was observed to support pigment production, further studies were conducted to optimize the concentration of YE required for maximal production of pigment. Results indicate that 3 g L^−1^ of YE supported maximum pigment production. The findings of El-Naggar and El-Shweihy [56], who utilized a basal medium consisting of YE, which considerably boosted the biomass and also the pigment content of *R. gelatinosus*, were in accordance with this finding. In another study, the utmost activity was shown in meat peptone containing medium 2 g L^−1^ for the red pigment production by *P. sinclairii* [57].

Under the previous optimal conditions, the significantly highest level of pigment formation by *Streptomyces* sp. LS1 strain was detected at level 8 g L^−1^ fructose. Budihal et al. [52] applied 1% starch for melanin production by *Streptomyces* sp. DSK2 and Gunasekaran and Poorniammal [57] applied 1.5% starch for pigment production by *Paecilomyces sinclairii*, while a low level of starch (0.2%) is optimum for the growth of melanin pigment producer *Streptomyces virginiae* as described by Deepthi and Rosamma [58].

To search out the optimal level of NaCl (the best salt), the previous experiment was repeated in presence of various levels of tested salt ranging (1-5 g L^−1^). The significantly highest level of pigment formation by studied LS1 strain was detected with level 3 g L^−1^ in presence of fructose (carbon source). At the lowest level of salt (1 g L^−1^), the smallest amount of pigmentation was shown. This observation was in agreement with some studies, where 2.5% of salinity increased the melanin production by *Vibrio cholera* [59]. It is known that hyperosmotic stress induces melanin production; this explained why melanin production occurred at a higher concentration of NaCl [59]. Also, Farkas and Monagha [60] reported that the best melanin production (3.4 mg mL^−1^) has been obtained at 2.5% of salinity. Yet, these differences between their results and ours related to the intraspecific variability and strain dependence

The antimicrobial activity of carotenoid pigment was studied against five species of marine bacterial pathogens of G_+ve_ (*B. subtilis* ATCC 6633 and *S. aureus* ATCC 6538) and G_−ve_ (*E. coli* ATCC 10418, *K. pneumoniae* ATCC 13883 and *P. aeruginosa* ATCC 9027) bacterial strains*.* The results indicated that G_−ve_ (*P. aeruginosa* ATCC 9027 and *K. pneumoniae* ATCC 13883) microorganisms were more susceptible to carotenoid pigment extracted from *Streptomyces* sp. LS1 strain than the G_+ve_ (*B. subtilis* ATCC 6633). This observation was in agreement with studies done by Manimala and Murugesan [61]. Fucoxanthin’s antibacterial activity on 13 aerobically grown bacterial strains was evaluated in another investigation. It was observed to have a significantly stronger impact on G_+ve_ than G_−ve_ bacterial strains [62]. The antifouling (AF) activity of the pigment was tested; it was noted the pigment has a remarkable valuable effect on biofouling reduction. This finding was in line with other studies with dried and fresh macroalgae *Chondrus crispus* (*Rhodophyceae*) crude ethanol extracts. To determine AF effectiveness, these extract was evaluated against five marine bacterial strains, five phytoplankton strains, and two macroalgae. Compared to the fresh source, the dried algae extract had a lower minimum inhibitory concentration (25μg mL^−1^) against the growth of bacteria and phytoplankton species than the fresh algal extract. The extracts were shown to have anti-germination activity against both *Undaria pinnatifida* and *Ulva intestinalis* spores in macroalgae tests, at a concentration of 25–50μg mL^−1^. The initial efficacy of AF paint with crude extract was found to persist for 6 weeks in a field study. The biocidal ability of photocatalytic TiO_2_-based nano compounds (also in combination with Ag and Cu nanoparticles) applied on travertine surfaces by spray-coating to limit or inhibit algal fouling. Stone’s aesthetic compatibility with colorimetry has been evaluated [63]. Antimicrobial and antifouling are mainly attributed to the synergistic effects of identified active compounds in the ethanolic extract as shown through GC-Ms starting from alcohol, phenol, fatty acids, and ester. These active compounds such as alcohol-related (2,3-Butanediol), phenolic-related compounds (Phenol, 2-methoxy-3-(2-propenyl)- and phenol, 2-methoxy-4-(2-propenyl)-, acetate), and fatty acid-related (vaccinic, hexadecanoic, octadecanoic) are characterized by several biological activities as potent antibacterial, antifungal, antiviral, and antioxidant.

## Conclusion

In the present study, *Streptomyces* sp. LS1 showed an ability to produce red pigment categorized as carotenoids based on spectroscopy and GC-MS analysis results. OVAT approach was followed for screening variables influencing the pigmentation bioprocess; further, the key variables were optimized. It was noticed during the optimization course that pigment production was systematically improved; both pigmentation and biomass production folded by 2.8 and 2.7 times, respectively. Throughout, the productivity yield reached 30mg of dried purified pigment/gram dry weight. The biological activities of the extracted pigment as antimicrobial and antifouling were demonstrated, where it showed a positive response against some marine bacterial pathogens and acted as anti-fouling towards marine microbes. Based on the results of this work, we can recommend the isolate LS1 as a good source of the natural bioactive red pigment to treat many human pathogens, especially G_−ve_ (*P. aeruginosa* ATCC 9027 and *K. pneumoniae* ATCC 13883), G_+ve_ (*S. aureus ATCC 6538*) and antifouling against marine microbes.

## Data Availability

All data produced during this study are included in this published article.
